# Efficient alignment of pyrosequencing reads for re-sequencing applications

**DOI:** 10.1186/1471-2105-12-163

**Published:** 2011-05-16

**Authors:** Francisco Fernandes, Paulo GS da Fonseca, Luis MS Russo, Arlindo L Oliveira, Ana T Freitas

**Affiliations:** 1 Instituto de Engenharia de Sistemas e Computadores: Investigação e Desenvolvimento (INESC-ID), R. Alves Redol 9, 1000-029 Lisboa, Portugal; 2 Instituto Superior Técnico-Universidade Técnica de Lisboa (IST/UTL), Av. Rovisco Pais, 1049-001 Lisboa, Portugal

## Abstract

**Background:**

Over the past few years, new massively parallel DNA sequencing technologies have emerged. These platforms generate massive amounts of data per run, greatly reducing the cost of DNA sequencing. However, these techniques also raise important computational difficulties mostly due to the huge volume of data produced, but also because of some of their specific characteristics such as read length and sequencing errors. Among the most critical problems is that of efficiently and accurately mapping reads to a reference genome in the context of re-sequencing projects.

**Results:**

We present an efficient method for the local alignment of pyrosequencing reads produced by the GS FLX (454) system against a reference sequence. Our approach explores the characteristics of the data in these re-sequencing applications and uses state of the art indexing techniques combined with a flexible seed-based approach, leading to a fast and accurate algorithm which needs very little user parameterization. An evaluation performed using real and simulated data shows that our proposed method outperforms a number of mainstream tools on the quantity and quality of successful alignments, as well as on the execution time.

**Conclusions:**

The proposed methodology was implemented in a software tool called TAPyR--Tool for the Alignment of Pyrosequencing Reads--which is publicly available from http://www.tapyr.net.

## Background

Sequencing by capillary electrophoresis, known as the Sanger method [1], has been employed in many historically significant large-scale sequencing projects and is regarded as the gold standard in terms of both read length and sequencing accuracy [2]. Several Massively Parallel DNA Sequencing (MPDS) technologies have recently emerged, including the Roche/454 GS FLX System, the Illumina/Solexa Genome Analyser, and the AB SOLiD System, which are able to generate a few orders of magnitude more bases per instrument run, being considerably less expensive than the Sanger method [2,3]. These technologies are enabling researchers and practitioners to efficiently sequence genomes, leading to very significant advances in biology and medicine. However, the huge volume of data produced by MPDS technologies creates important computational challenges [4]. Moreover, the different platform-specific data characteristics require different algorithmic approaches. For instance, some applications may use the 454 Titanium platform to produce reads 400 bases long, some other studies may employ a SOLiD system set to produce short reads of 35 bases, and yet other projects may use the Illumina system to produce 2 × 75 bases paired-end reads. Given their large variety, it would be rather difficult for a single algorithm to handle all kinds of data optimally.

When sequencing a new organism, one is usually faced with the problem of assembling the sequence fragments (reads) together from scratch. However, when a sufficiently close sequence is already known, one may choose to use it as a reference and proceed by first mapping the reads to this reference and then determining the new sequence by extracting the consensus from the mapping results. The former strategy is called *de novo *sequencing, while the latter is known as *re-sequencing*. Several tools have recently been developed for generating assemblies from short reads, e.g [5,6]. Similarly, several methods have been proposed to address the problem of efficiently mapping MPDS reads to a reference sequence, like [7-12], to cite a few. As referred before, the sheer volume of data generated by MPDS technologies (to the order of hundreds of gigabases per run), and the need to align reads to large reference genomes limit the applicability of standard techniques. Indeed, in a typical application, we may have to align hundreds of millions of reads to a reference genome that can be as large as few gigabases, a job that cannot be efficiently achieved through standard dynamic programming procedures.

One way to speed up the read alignment task is to resort to approximate indexing techniques. A first generation of aligners was based on hash tables of *k*-mers. Some of them, like SSAHA2 [13], build tables of *k*-mers of the target sequence, whilst others, like Newbler [14], index the reads, thus presumably requiring re-indexing for each new run. Recent developments in the field of compressed approximate indexes have led to a new family of alignment algorithms such as Segemehl [10], which uses an enhanced suffix array (see Implementation), and BWA-SW [11], which uses a FM-index (see Implementation) to accelerate Smith-Waterman alignments. Yet the number of aligners that support GS FLX pyrosequencing data is, as of today, relatively scarce compared to other technologies, most notably Illumina. Moreover, some of these tools find their origins in the days before the advent of the new sequencing technologies and only later were adapted to cope with new kinds of data [13], and some others target multiple kinds of data [10] being not optimized for pyrosequencing data. Given this state of affairs, we argue that there is still room for improvement in the realm of publicly available aligners specifically designed for high-throughput pyrosequencing data.

In this paper we present a new method for the alignment of pyrosequencing reads, like those produced by the 454 GS FLX platform. By focusing on this specific technology, our procedure manages to explore its data characteristics to achieve improved performance over other mainstream methods. Like many of those methods, ours also builds an index of the target (reference) sequence to accelerate the alignment. It then employs a multiple seed heuristic to anchor the best candidate alignments. Contrary to other seed-based alignment tools, our strategy adds more flexibility by dispensing with the need of determining the number and length of the seeds beforehand. Our heuristic relies on some assumptions that can be reasonably expected to hold true for re-sequencing projects based on pyrosequencing data, namely, that the optimal alignments are mostly composed of relatively large chunks of exact matches interspersed by small, possibly gapped, divergent regions. A banded dynamic programming is used to finish up the candidate multiple seed alignments considering user-specified error constraints. A detailed description of the algorithm and data structures is given in the "Implementation" section. In the "Results and Discussion" section, we present a comparison between our method and a set of tools of widespread use for the local alignment of pyrosequencing reads. We base our discussion on results obtained with both real and simulated data.

## Implementation

### Compressed indexes

**Figure 1:**
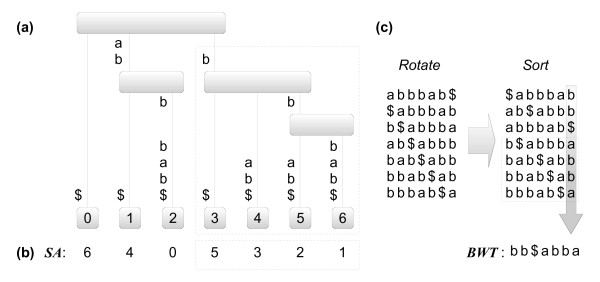
**Indexes for the text *abbbab***. (a) Suffix tree for the text *abbbab*, with the leaves numbered according to lexicographic order of the suffixes they represent. (b) The corresponding suffix array indicating the starting position of the sorted suffixes. (c) The equivalent BWT obtained by rotating and then sorting the text. Notice that the BWT is actually composed of only the last letter of the sorted rotations. Interestingly, this representation permits reconstructing the original text, and is also much more amenable to compression because it typically contains sequences of repeated characters. Notice, in addition, that every subtree of the suffix tree corresponds to an interval of the suffix array and, equivalently, of the BWT. In this example, the dashed boxes indicate the subtree/intervals corresponding to the suffixes that start with the common prefix *b*.

The main data structure for sequence pattern matching is an index. Indexes reduce the time for matching a pattern because they restrict the search to the positions where it may occur instead of scanning the whole text. One of the most, if not the most, popular index structures is the *suffix tree *(ST), which is obtained by identifying common prefixes of the different suffixes of the represented text to nodes of a tree (see Figure 1(a) for an example). In such a structure, a pattern can be searched following edges with matching labels down from the root. Each leaf of the suffix tree represents a suffix of the text and, more generally, each node represents the subset of suffixes corresponding to the leaves of the subtree rooted at that node. The downside of indexes is that they need to be constructed *a priori *and have a bad reputation of using too much space. Despite providing for fast searching algorithms, suffix trees are particularly known for this bad characteristic. A popular alternative to suffix trees are *suffix arrays *(SA), that require asymptotically the same space, *O*(*n*) computer words for a text of size *n*, but with a smaller proportionality factor. Suffix arrays are obtained by ordering the suffixes of a text lexicographically. A correspondence can be established between nodes of the suffix tree and contiguous intervals of the suffix array. An example of a suffix array is shown in Figure 1(b). Detailed descriptions of string matching and indexes, including the ones mentioned here, are widely available [15].

Recent research on indexes has focused on the fact that pointer representations require *O*(*n *log *n*) bits whereas the original text (the target genome, in our case) requires only *n *log *σ *bits, where *σ *is the alphabet size, e.g. 4 for DNA and 20 for proteins. In an effort to reduce this gap, new indexes have been designed which became collectively known as *compressed indexes *[16] due to the fact that they rely heavily on data compression techniques. In spite of their reduced space, compressed indexes can be made to allow for an even broader range of operations than classical indexes, like generalized branching, that combines blocks of letters instead of just one letter at a time [17]. Our method uses an implementation of the FMIndex [18] optimized for the DNA alphabet. The FMIndex is a compressed index based on the Burrows-Wheeler transform (BWT) [19] requiring only *O*(*n *log *σ*) bits of memory space. The BWT of a text *t *is obtained by appending an extra symbol $ to *t*, and then sorting all cyclic permutations (rotations) of *t*$ according to the lexicographic order, with $ being the lowest symbol. Thus a BWT of a text is essentially equivalent to its suffix array. In fact, they are related through the formula BWT[*i*] = *t*[SA[*i*] - 1], for *i *= 1, . . . , | *t *|. An example of a BWT is given in Figure 1(c).

### The seed-based search approach

Due to the relatively large size of the GS FLX reads, it is not practical to use a plain index-based exact matching algorithm. Some sort of backtracking strategy could be used to allow for errors but, since the number of possible comparisons increases exponentially with their number, this becomes rapidly inefficient. Instead, we propose a seed-based search heuristic that explores the characteristics of the pyrosequencing data and of the *bona fide *alignments that are likely to arise in the context of re-sequencing applications. Since the error rates are usually low [20], and the prevalent type of pyrosequencing errors are small indels, with mismatches being much less common, we conjecture that the optimal alignments are expected to be formed of large chunks of exact matches interspersed by divergent gapped regions. Moreover, since the read lengths are of a few hundred bases, we can expect the exact match regions to be large enough so we can use segments of them, called *seeds*, as a backbone to pin down the position of the alignment on the reference sequence, or at least reduce the amount of candidate positions to a manageable number of possibilities that can be tested individually. In this case, the optimal alignments can be obtained by expanding the candidate multiple seed matches into alignments of the whole read by filling up the remaining regions and selecting those with best overall scores.

Our strategy for choosing the seeds consists in approximately partitioning the read into maximal exact match blocks in a greedy fashion. More precisely, let *r *= *r*_1 _⋯ *r_m _*be the read. The procedure starts at the first position of the read and uses the index to find the largest prefix of the read with exact occurrences in the reference sequence, say *r*[1 ⋯ *l*] = *r*_1 _⋯ *r_l_*. In practice we obtain the equivalent of an interval of the BWT which contains the positions of the reference sequence *g *at which *r*[1 ⋯ *l*] occurs. Obviously, by maximality, *r*[1 ⋯ *l *+ 1] does not occur in *g*. This happens because none of the occurrences of *r*[1 ⋯ *l*] is followed by *r*_*l*+1 _or, put another way, because there is a mismatch between *r*_*l*+1 _and the letter following each occurrence of *r*[1 ⋯ *l*] (or because it occurs at the very end of *g*). If *r*_*l*+1 _≠ *r_l_*, then we set *r*[1 ⋯ *l*] as the first seed and proceed as above to find the next seed starting from position *l *+ 2. If, however, we have *r*_*l*+1 _= *r_l_*, this means that the difference occurred in the middle of a homopolymer (contiguous subsequence of identical bases), most likely due to an insertion sequencing error. In this case, we set *r*[1 ⋯ *l*] as the first seed as before, but advance the cursor to the start of the next homopolymer, i.e. to the smallest *l*' >*l *s.t. *r*_*l*' _≠ *r*_*l*' _- _1_. We repeat this process until the end of the read is reached.

Once we have the set of seeds and their individual positions in the reference sequence, we need to identify subsets of occurrences of distinct seeds that are in accordance with their original order and spacing in the read, which can then serve as a support for the final alignments. More formally, let *g *be the reference sequence, and *r *be a read. Let also *s*_1_, . . . , *s_k _*be *k *substrings of *r *such that *r *= *s*_1_*a*_1_*s*_2_*a*_2 _⋯ *a*_*k*-1_*s_k_a_k_*, where, for *i *= 1, . . . , *k*, *s_i _*denote the seeds and *a_i _*denote the substrings in between them. For each *i *= 1, . . . , *k*, let *o_i _*≥ 0 be the number of exact occurrences of *s_i _*in *g*. We then have  ways to choose a set containing one occurrence for each of the *k *substrings. Let *p *= (*p*_1_, . . . , *p_k_*) be one of such *o *tuples of distinct seed occurrences. If, for *i *= 1, . . . , *k -*1, we have *p_i _*≤ *p*_*i*+1 _and |*a_i_*| ∈*_i _p*_*i*+1 _- (*p_i _*+ |*s_i_*|) |*a_i_*| + ∈*_i _*for some given ∈*_i _*≥ 0, then we say that these occurrences are *coherent*. Hence, a coherent set of seed occurrences is composed of positions which respect the relative order of the corresponding seeds in the read and such that, for any two consecutive seeds, the distance between their occurrences lies within a restricted interval around the actual distance between those seeds in the read. For the sake of flexibility, we do not restrict ourselves to coherent sets containing occurrences of all the seeds. We also take partial sets containing occurrences of only some of those seeds as good candidates for further expansion.

The set of seeds (*s*_1_, . . . , *s_k_*) and their occurrence positions in *g *can be obtained with the index in linear time. However, the number of combinations of occurrences of different seeds can be rather large, especially if some of them are short. This makes it impractical to test all possibilities for coherence. Nonetheless, most of these combinations will typically be non-coherent, and if we care to previously sort the set of occurrences of each seed, we can efficiently search for coherent combinations using, again, a greedy strategy, simply by scanning the seed matches in *g *from left to right, partitioning them into maximal non-overlapping sets of consecutive coherent matches. Although this might seem a rough approach at first glance, in fact this strategy has shown to be adequate because of the relatively large size of the seeds and small separation between them, which makes it difficult for occurrences of two consecutive seeds to be interspersed with an occurrence of a third one.

**Figure 2:**
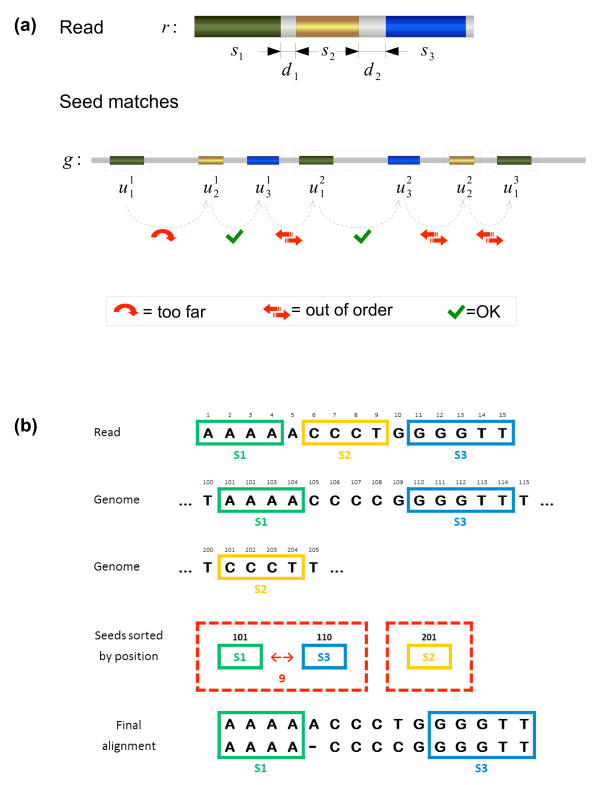
**Sketch and example of the TAPyR procedure**. (a) Sketch of the seed strategy employed by TAPyR. In this schema, three seeds are chosen, and seven matches of these seeds are found on the reference genome, three for the first seed, two for the second, and two for the third. These occurrences are ordered in the genome and scanned from left to right. Multiple seed matches are formed by extending current partial matches with the next occurrence if the coherence criteria are met. Otherwise, the current multiple match is stored as a potential candidate and a new one is started. In this example, we finish with five potential candidates for extension indicated by the dashed boxes. The largest candidate(s), i.e. the multiple seed occurrence that span most bases, are chosen for extension. In this case, that should be . (b) A more concrete example, in which we have a sequence of length 15, which was originally read from position 101 of the genome, with one insertion at position 5 and one substitution at position 9. The algorithm starts searching from the beginning of the read in the index, but cannot continue beyond the fourth *a *character. At this point, we have the first seed *s*_1 _= *aaaa*, which occurs at position 101 in the genome. The next character of the read is skipped, and the search continues from position 6, which is the beginning of the second seed. Seed *s*_2 _= *ccct *happens to have an accidental occurrence at the position 201, which is not related to the actual read position in the genome. Again, we skip the next (mismatched) character of the read and restart at position 11. This time the search reaches the end of the read, and yields the last seed *s*_3 _= *gggtt*, occurring at position 110. These three occurrences are now sorted according to their position in the genome, and it turns out that the occurrences of *s*_1 _and *s*_3 _form a coherent multiple seed occurrence of combined length 9. The other candidate would be composed of the occurrence *s*_2 _alone which is not chosen for expansion since it is smaller. The space between the two seeds is then filled using dynamic programming, and the correct mapped position (101) is returned along with the final alignment.

Once the potential read occurrences indicated by coherent multiple-seed matches are found, the algorithm runs a banded Needleman-Wunsch dynamic programming procedure with Gotoh's modifications to align the non-seed segments of the read to their counterparts in the genome. That is, if we have a coherent set of occurrences of the seeds *s*_*j*1_, . . . , *s*_*jq *_, s.t. the read decomposes into *r *= *b*_0_*s*_*j*1_*b*_1_*s*_*j*2 _*b*_2 _*b*_*q*-1_*s_jq _b_q_*, and the reference sequence into *g *= *c*_0_*s*_*j*1_*c*_1_*s*_*j*2 _*c*_2 _⋯ *c*_q __1_*s_jq_c_q_*, then we align each pair (*b_i_*, *c_i_*), for *i *= 0, . . . , *q*. Of course, for both ends, (*b*_0_, *c*_0_) and (*b_q_*, *c_q_*), we perform semi-global alignments. The largest candidate coherent multiple seed matches are extended this way and accepted as a read occurrence if either the overall alignment identity stays above a given threshold percentage *t *or, alternatively, if the sum of the errors in-between the seeds and at the extremes of the read do not exceed a pre-established number *e*. The algorithm can be chosen to report all the accepted occurrences or only the one(s) with the least errors. The strategy described above is illustrated in Figure 2.

### Synthetic data generation

In order to evaluate the algorithms in a controlled setting, we generated artificial data sets with a procedure inspired by empirical studies on GS FLX data [20,21], and designed to yield reads with characteristics similar to real data. In our procedure, *n *random contiguous subsequences are extracted from a given 'source' sequence *g*. The lengths of these initial subsequences are drawn from a normal distribution with mean *μ_l _*and standard deviation *σ_l_*, also provided as input. Next, these subsequences are modified to simulate sequencing errors as follows. In the GS FLX high-throughput pyrosequencing procedure, the template molecules are sequenced one maximal homopolymer at a time (formally, a homopolymer can consist of a single base), as opposed to one base at a time in the traditional Sanger method. Hence, the most common type of error in pyrosequencing consist in the misinterpretation of the intensity of the signal that determines the length of the homopolymer being read, leading to an insertion or deletion of identical consecutive bases in the read, relative to the actual template sequence. Miscalled base errors (substitutions) also occur but they are comparatively much less frequent. Sequence quality is known to be non-uniform along the read, being lower at the extremes, particularly towards the 3' end. Also, errors tend to affect long homopolymers more than short ones. However, for the sake of simplification, we consider that errors are uniformly distributed along the read and that the prevalence and size of indels are not affected by the length of the homopolymers. More precisely, the procedure takes in three parameters *p*_sub_, *p*_ins _and *p*_del _which correspond to the probabilities of having a substitution, an insertion or a deletion in any given homopolymer, regardless of its length and position in the read. Moreover, these events are considered to be mutually exclusive, that is, we assume that for any homopolymer being sequenced, there can either be a substitution error with probability *p*_sub_, an insertion with probability *p*_ins_, a deletion with probability *p*_del _or it can be correctly sequenced with probability 1 - (*p*_sub _+ *p*_*i*ns _+ *p*_del_). Whenever a mismatch takes place, the miscalled base is randomly chosen according to substitution probabilities indicated in a matrix *m*, given as input. Each row/column of *m *corresponds to a nucleotide and the element *m*[*a*, *b*] indicates the probability for *a *to be miscalled as (replaced by) *b*. As for indels, the lengths of the gaps are drawn from Zipfian distributions, which are discrete power-law distributions with mass function . In our case, *ω *is a positive integer parameter that corresponds to a maximum allowed gap size, and *γ *> 0 controls the shape of the distribution: the greater its value the higher the prevalence of small gaps. We use specific exponent parameters, *γ*_ins _and *γ*_del_, for insertion and deletion operations, respectively.

## Results and Discussion

We evaluated TAPyR against other mainstream mapping tools which are also able to deal with high-throughput pyrosequencing reads, namely BWA-SW [11], SSAHA2 [13], Segemehl [10], GASSST [12], and Newbler [14]. Our analyses were performed with real and simulated data sets, with the objective of assessing the efficiency and accuracy of the aforementioned tools in the context of re-sequencing projects.

### Results on real data

The biological data sets we used, summarized in Table 1, encompass a reasonable variety of organism types, including two bacteria (*Streptococcus pneumoniae *and *Escherichia coli*), one protozoan (*Plasmodium falciparum*), one nematode (*Caenorhabditis elegans*), one insect (*Drosophila pseudoobscura*), and one human chromosome. They also cover re-sequencing applications with reads from individuals of the same species (human), different and mutated strains of the same species (bacteria and worm), and different (sub-)species (fly).

**Table 1:** Real data sets

Reference genome Source	Reference genome size	SRA accession and species	Total reads	Average read length
*S. pneumoniae* ATCC 700669 [GenBank: FM211187]	≈2.2 Mbp	○ SRR001327 *S. pneumoniae *CDC1873-00 ○ SRR001328 *S. pneumoniae *SP195 ○ SRR001329 *S. pneumoniae *CDC0288-04	646,724	253

*E. coli *0127:H6 E2348/69 [GenBank: FM180568.1]	≈4.96 Mbp	○ SRR000868 *E. coli *K-12 ○ SRR000870 *E. coli *K-12 ○ SRR031369 *E. coli *ETEC WS3080A ○ SRR031370 *E. coli *ETEC TW03576	588,397	263

*P. falciparum *3D7 PlasmoDB rel 7.0	≈23.3 Mbp	○ SRR006911 *P. falciparum *3D7 ○ SRR006912 *P. falciparum *3D7 ○ SRR006913 *P. falciparum *3D7 ○ SRR006914 *P. falciparum *3D7 ○ SRR006915 *P. falciparum *3D7	203,196	223

*C. elegans* WormDB rel. WS210	≈103 Mbp	○ SRR022943 *C. elegans *Lynch MA41 mutation-accumulation line derived from N2.	3,214,353	103

*D. pseudoobscura *FlyBase rel. 2.14	≈150 Mbp	○ SRR003807 *D. pseudoobscura *Flagstaff 1993 ○ SRR014458 *D. pseudoobscura bogotana *ER (white) ○ SRR014459 *D. pseudoobscura bogotana *ER (white) ○ SRR014460 *D. miranda *strain Mather 1993	834,659	239

*H. sapiens *Chr. 15 ENSEMBL ver. GRCh37	≈100 Mbp	○ SRR014420 Human individual NA15510 ○ SRR014421 Human individual NA15510 ○ SRR014422 Human individual NA15510 ○ SRR014423 Human individual NA15510 ○ SRR014424 Human individual NA15510 ○ SRR014425 Human individual NA15510	3,204	212

Biological data sets used for the evaluation of the algorithms. The read data sets were downloaded from the Sequence Read Archive (SRA) public repository.

In this experiment, we wanted to analyze the ability of the algorithms to produce high coverage mappings, which directly relates to the proportion of reads that can be successfully mapped. High coverage is essential to the successful completion of a re-sequencing project, with about 20-25× coverage being required for optimal results with the GS FLX technology [22]. Attaining such high levels depends naturally on the amount of available data, but equally on the capacity of the alignment tool to map the reads correctly, especially in the presence of inevitable sequencing errors and natural variation. The other aspect we wanted to assess was the efficiency of the algorithms in terms of computation time. Efficiency is a critical aspect for any algorithm in modern high-throughput data processing pipelines, given the rapid increase in the volume of data being produced.

The results of our tests are shown in Table 2. In that comparison, we included two lines corresponding to TAPyR being set to report alignments with at least 50% (TAPyR 50) and 85% (TAPyR 85) identity. These illustrative values match the default options of other tools: 85% for Segemehl, and 50% for SSAHA2. As can be seen, in almost all direct comparison scenarios, TAPyR has shown to be several times faster than the other tools. As for the number of successfully aligned reads (the "% reads" columns), we notice first that the other algorithms display quite similar figures, with no tool consistently aligning more reads that the others. With the minimum identity threshold set to 85% (TAPyR 85), our method aligns a smaller quantity of reads. However, if we investigate the number of errors (gaps and mismatches) of the reported alignments by computing the ratio between the number of base-pair matches and the number errors (the "bp/err" columns), we see that TAPyR is using a more conservative heuristic which tends to produce alignments of a higher identity level at the expense of dropping a slightly larger number of reads. Indeed, if we lower minimum identity requirement to 50% (TAPyR 50), then our tool aligns more reads than all the others in all data sets at comparable average error rates, and with a minimal time overhead.

**Table 2:** Experimental results with real data

	*S. pneumoniae*			*E. coli*			*P. falciparum*		
	*time*	% *reads*	*bp/err*	*time*	% *reads*	*bp/err*	*time*	% *reads*	*bp/err*
BWA-SW	19m	92.86	40	16m	65.4	22	8m	95.98	56
SSAHA2	6m	92.86	41	5m	65.4	23	68m	98.45	57
Newbler	11m	92.95	47	11m	65.55	24	12m	97.72	60
Segemehl	181m	90.77	61	110m	62.95	32	90m	98.14	62
GASSST	6m	89.96	63	4m	62.71	32	3m	62.80	72
TAPyR 85	2m	88.37	70	13m	59.98	35	48s	95.30	66
TAPyR 50	3m	93.33	40	20m	66.80	20	51s	98.91	53

	***C. elegans***			***D. pseudoobscura***			***H. sapiens***		
	***time***	**% *reads***	***bp/err***	***time***	**% *reads***	***bp/err***	***time***	**% *reads***	***bp/err***

BWA-SW	51m	61.05	19	58m	95.80	19	6s	98.25	58
SSAHA2	513m	70.40	18	92m	97.30	18	16s	99.44	54
Newbler	45m	69.07	19	119m	96.86	23	1m	98.72	78
Segemehl	249m	68.10	23	339m	90.98	28	52s	96.41	89
GASSST	9m	58.50	27	22m	82.80	30	1m	83.61	93
TAPyR 85	31m	55.59	35	7m	85.91	30	1s	95.13	96
TAPyR 50	31m	73.30	15	7m	97.05	19	2s	99.63	60

Results of the experiments performed with real biological data. These tests were run on a Linux server with 16 Gb of RAM. TAPyR was tested under two configurations, requiring alignments with at least 50% and 85% identity, designated by TAPyR 50 and TAPyR 85, respectively. The other tools were run with their default options for 454 data, except for the following modifications. SSAHA2 and Segemehl were set to report only the best alignment for each read. For GASSST, we set the minimum identity to 85% (option -p 85) to match Segemehl. Newbler was set not to generate large files (option -nobig) and to load the index into the main memory (option -m). Reported times refer to the total number of CPU-seconds that the process used directly, as given by the Linux command time -f "%U". The average base-pairs-per-error rates ("*bp*/*err*" columns) are computed based on the best reported alignment of each read only.

### Results on synthetic data

We also performed tests using simulated data produced according to the procedure described in the Methods section. We generated three data sets of *N *= 300,000 synthetic reads from the 250 Mbp sequence of the human chromosome 1 [GenBank: NC_000001.10]. These data sets are supposed to mimic the data obtained in a typical run of the GS FLX instrument at different sequencing error levels. Hence, the first data set, hereafter referred to as HS1, was generated with the read generator parameters set as *μ_l _*= 250, *σ_l _*= 50, *p*_ins _= *p*_del _= *p*_sub _= 0.01, *ω *= 10, *γ*_ins _= *γ*_del _= 3, and equiprobable substitution rates *M*[*a*, *b*] = 1/3, for *a *≠ *b*. In this setting, we have a 1% chance of each of the three kinds of error when reading a homopolymer. For the second data set, HS2, we increased the error levels of each kind to 5% by setting *p*_ins _= *p*_del _= *p*_sub _= 0.05, and for the third data set, we added a considerable amount of noise by setting *p*_ins _= *p*_del _= *p*_sub _= 0.10 (only 70% of chance for each homopolymer to be sequenced correctly). The purpose of this experiment was mainly to test accuracy of the procedures by computing the fraction of reads mapped back to their original positions, as well as to assess the robustness of the heuristic to different error levels.

The results of the tests are shown in Table 3. We notice that all algorithms give quite accurate results in all the tested conditions. In any case, TAPyR behaved among the best in terms of accuracy, mapping virtually all reads correctly, showing thus resilience to noise up the tested levels. Moreover, as in the previous experiments, our method has confirmed to be fastest by a comfortable margin.

**Table 3:** Experimental results with synthetic data

	HS1		HS2		HS3	
	*time*	% *accuracy*	*time*	% *accuracy*	*time*	% *accuracy*
BWA-SW	1358s	99.32	1269s	98.81	1212s	94.10
SSAHA2	5044s	99.86	4328s	99.86	4121s	99.18
Newbler	1192s	99.99	6066s	95.53	7260s	83.25
Segemehl	3824s	99.99	6823s	99.95	6299s	98.81
GASSST	855s	99.47	794s	99.27	693s	97.35
TAPyR 50	63s	99.99	113s	99.78	239s	98.20

Results of the experiments performed with synthetic data. The data sets were generated as described in the text. The tests were run under the same conditions as those with real data. Shown is the percentage of reads of each data set that were successfully aligned to their original positions in the reference sequence.

### Memory usage

We also measured the memory requirements of the evaluated tools in the tests with real data discussed above. The figures presented in Table 4 show the sizes of the index files on disk, when they exist, and the peak usage of main memory for the different data sets. As it can be seen, BWA displayed the smallest requirements in absolute terms, followed closely by TAPyR. The other tools, especially those based on *k*-mer tables, demand substantially more memory. As expected, TAPyR's index files scale linearly with the size of the indexed genomes (by a multiplicative factor of ≈ 1.6). Apart from the index, which is loaded into main memory, TAPyR uses only a small additional amount of space (mainly for the dynamic programming part), so that the total amount of required RAM also scales linearly with the indexed genome (by a factor of ≈ 2.5). These modest and predictable requirements make TAPyR suitable for large genomes with moderately-sized machines.

**Table 4:** Memory requirements for real data

	*S. pneumoniae*		*E. coli*		*P. falciparum*	
	*index(Mb)*	*RAM(Mb)*	*index(Mb)*	*RAM(Mb)*	*index(Mb)*	*RAM(Mb)*
BWA-SW	3	27	7	29	34	59
SSAHA2	517	788	521	773	552	717
Newbler	n/a	600	n/a	900	n/a	500
Segemehl	31	302	68	328	315	447
GASSST	n/a	2381	n/a	2479	n/a	2539
TAPyR 50	4	7	8	18	39	64

	***C. elegans***		***D. pseudoobscura***		***H. sapiens***	
	***index(Mb)***	***RAM(Mb)***	***index(Mb)***	***RAM(Mb)***	***index(Mb)***	***RAM(Mb)***

BWA-SW	144	149	210	208	118	103
SSAHA2	679	1559	755	1209	648	680
Newbler	n/a	5100	n/a	2300	n/a	500
Segemehl	1388	2439	2025	2643	1134	1299
GASSST	n/a	5657	n/a	6967	n/a	4752
TAPyR 50	168	270	244	394	137	220

Memory requirements for the real biological data sets of Table 1. Shown are the index file size (when applicable) and peak main memory usage as measured by the tool tstime

http://bitbucket.org/gsauthof/tstime, except for Newbler, whose overall memory demand was estimated through the Linux htop tool.

## Conclusions

The combination of state of the art indexing techniques and a seed-based search approach led to the development of a new read mapping method for high-throughput pyrosequencing data. By using an effective heuristic which explores the characteristics of this particular kind of data in the context of typical re-sequencing applications, our method manages to achieve convincing performance in terms of speed and in terms of the number and precision of aligned reads, as demonstrated by our tests with real and simulated data. In fact, our proposed solution has displayed class-leading CPU-time performance and excellent use of input reads in comparison to other mainstream tools. An added-value of our procedure comes from the fact that it requires almost no external parameterization. As a matter of fact, the main user options are end-of-the-chain cutoff parameters that concern the quality of the reported alignments in terms of minimal identity or maximal number of errors, having no consequence on the accuracy of the heuristic and only marginal impact on the overall execution time. Memory requirements are also on par with the best in this category of tools, being not only small in absolute terms but, more importantly, linearly proportional to the size of the input reference sequence by a small factor. Based on these results, we propose that TAPyR constitutes an advantageous alternative for re-sequencing projects based on pyrosequencing data.

## Availability and requirements

Project name: TAPyR--Tool for the Alignment of Pyrosequencing Reads

Project home page: http://www.tapyr.net

Operating system(s): multiple (requires a C compiler only)

Programming language: C

Other requirements: none (see Results section for an idea of memory usage)

License: GNU GPL

Restrictions to use by non-academics: none additional

## Authors' contributions

AF and AO conceived the project. All authors have participated in the design and refinement of the method and in the analysis of the results. FF wrote most of the code, helped by LR and PF. FF and PF collected the data and performed the experiments. The authors collectively drafted the manuscript. PF, AF, and FF wrote most of the text. All authors revised and approved the final version.
